# Supplementary material for: Effects of a social support family caregiver training program on changing blood pressure and lipid levels among elderly at risk of hypertension in a northern Thai community
Source: PLoS One. 2021 Nov 30;16(11):e0259697. doi: 10.1371/journal.pone.0259697 (PMC8631627; doi:10.1371/journal.pone.0259697)
Supplement: S1 File — (DOCX) [file pone.0259697.s001.docx]

## **แบบสัมภาษณ์สำหรับงานวิจัย**

#### ส่วนที่ 1 แบบสัมภาษณ์ข้อมูลส่วนบุคคล (ผู้สูงอายุ)

**คำชี้แจง** แบบสัมภาษณ์ข้อมูลทั่วไปของผู้ตอบแบบสัมภาษณ์ โปรดใส่เครื่องหมาย (/) ลงในช่องสี่เหลี่ยมเพียงตัวเลือกเดียวหรือเติมคำตอบลงในช่องว่างตามความเป็นจริง

| **คำถาม** | **รายละเอียด** |
| --- | --- |
| 1. อายุ | ……………………………………….ปี |
| 2.เพศ | 1) ชาย 2) หญิง |
| 3. สถานภาพสมรส | 1) โสด 2) คู่  3) หม่าย 4) หย่า 5) แยกกันอยู่ |
| 4. ระดับการศึกษา | 0) ไม่ได้รับการศึกษา 1) ประถมศึกษา  2) มัธยมศึกษา 3) ปริญญาตรี  4) สูงกว่าปริญาตรี 5) อื่น ๆ โปรดระบุ……………………….. |
| 5. ความเพียงพอของรายได้ | 0) ไม่เพียงพอ 1) เพียงพอ   3) อื่น ๆ โปรดระบุ……………………….. |
| 6. รสชาอาหารที่ท่านชอบรับประทาน | 0) ไม่มี 1) เค็ม  2) หวาน 3) มัน   4) เปรี้ยว 5) อื่นๆ (ระบุ)…….................... |
| 7. ปัจจุบันทานออกกำลังกาย/มีกิจกรรมทางกายหรือไม่ | 0) ไม่ออกกำลังกาย  1) มี เดินรอบบ้านทุกวัน  1) มี งานบ้านทุกวัน  1) มี ออกกำลังกาย (อย่างน้อยวันละ2-3 ครั้ง/สัปดาห์)  1) ทำ เดินรอบบ้านทุกวัน |
| 8. ปัจจุบันท่านดื่มเครื่องดื่มแอลกอฮอล์หรือไม่ | 0) ไม่ดื่ม 1) ดื่ม |
| 9. ปัจจุบันท่านสูบบุหรี่หรือไม่ | 0) ไม่สูบ 1) สูบ |
| 10. ปัจจุบันดัชนีมวลกายของท่าน | โปรดระบุ ............................................kg/m^2^  เทียบระดับ  1.1) ผอม (< 18.5) 1.2) ปกติ (18.5 – 22.90)  1.3) น้ำหนักเกิน (23 – 24.90) 1.4) โรคอ้วนระดับที่ 1 (25 – 29.90 )  1.5) โรคอ้วนระดับที่ 2 (≥30) |
| 11. การได้รับข้อมูลข่าวสารเรื่องโรคความดันโลหิตสูง | 0) ไม่รู้  1) รู้  1.1) เจ้าหน้าที่ทางการแพทย์และสาธารณสุข 1.2) อสม.  1.3) สื่อออนไลน์ เช่น เฟสบุ๊ค ไลน์ 1.4) บุคคลในครอบครัว  1.5) อื่นๆ ระบุ................. |
| 12. ค่าคอเลสเตอรอล  (Total cholesterol) | ............................................................................ mg/dl |
| 13. ระดับความดันโลหิต | ............................................................................ mmhg |

**สำหรับผู้ดูแล/ญาติ**

**คำชี้แจง** แบบสัมภาษณ์ข้อมูลทั่วไปของผู้ตอบแบบสัมภาษณ์ โปรดใส่เครื่องหมาย (/) ลงในช่องสี่เหลี่ยมเพียงตัวเลือกเดียวหรือเติมคำตอบลงในช่องว่างตามความเป็นจริง

| **คำถาม** | **รายละเอียด** |
| --- | --- |
| 1. อายุ | ……………………………………….ปี |
| 2.เพศ | 1) ชาย 2) หญิง |
| 3. สถานภาพสมรส | 1) โสด 2) คู่  3) หม้าย 4) หย่า 5) แยกกันอยู่ |
| 4. ระดับการศึกษา | 0) ไม่ได้รับการศึกษา  1) ประถมศึกษา 2) มัธยมศึกษา  3) ปริญญาตรี 4) ปริญญาตรีหรือสูงกว่า  5) อื่น ๆ โปรดระบุ……………………….. |
| 5. ปัจจุบันท่านประกอบอาชีพใด | 1) ไม่ได้ประกอบอาชีพ  2) ประกอบอาชีพ  2.1) รับจ้าง 2.2) เกษตรกร  2.3) ค้าขาย 2.4) เกษียณอายุราชการ   2.5) อื่น ๆ โปรดระบุ……………………….. |
| 6. ความเพียงพอของรายได้ | 0) ไม่เพียงพอ 1) เพียงพอ   2) อื่น ๆ โปรดระบุ……………………….. |
| 7. ท่านมีสัมพันธภาพอย่างไรกับผู้สูงอายุ | 0) ไม่รู้จัก 1) สามี หรือ ภรรยา  2) ลูกในสายเลือด  3) หลาน/เหลน  4) ญาติพี่น้อง 5) คนข้างบ้าน  6) อสม. 7) อื่นๆ (ระบุ)…….................... |
| 8. ปัจจุบันท่านดื่มเครื่องดื่มแอลกอฮอล์หรือไม่ | 0) ไม่ดื่ม 1) ดื่ม |
| 9. ปัจจุบันท่านสูบบุหรี่หรือไม่ | 0) ไม่สูบ 1) สูบ |
| 10. การได้รับข้อมูลข่าวสารเรื่องโรคความดันโลหิตสูง | 0) ไม่รู้  1) รู้  1.1) เจ้าหน้าที่ทางการแพทย์และสาธารณสุข 1.2) อสม.  1.3) สื่อออนไลน์ เช่น เฟสบุ๊ค ไลน์ 1.4) บุคคลในครอบครัว  1.5) อื่นๆ ระบุ................. |

**ส่วนที่** **2 แบบสัมภาษณ์ความรู้เรื่องโรคความดันโลหิตสูง**

**คำชี้แจง** โปรดทำเครื่องหมาย 🗸ลงในช่องว่าง ที่ตรงตามความรู้สึก ความคิดเห็น ตามความเชื่อของท่านในข้อต่อไปนี้เพียงช่องเดียว คำตอบของท่านไม่มีการตัดสินว่าผิดหรือถูก

| **คำจำกัดความ** |  |  |  |
| --- | --- | --- | --- |
| (1) | ใช่ | หมายถึง | ท่านไม่แน่ใจกับข้อคำถาม |
| (0) | ไม่ใช่ | หมายถึง | ท่านไม่เห็นด้วยกับข้อคำถาม |

| **ข้อที่** | **ข้อความ** | **ใช่** | **ไม่ใช่** |
| --- | --- | --- | --- |
| 1 | เป็นโรคความดันโลหิตสูงแล้วไม่รักษา จะทำให้สายตาเสื่อม |  |  |
| 2 | โรคความดันโลหิตสูงอาจจะทำให้เป็นโรคไตวาย |  |  |
| 3 | โรคความดันโลหิตสูง มีส่วนทำให้เกิดเส้นเลือดแดงใหญ่ผิดปกติ |  |  |
| 4 | ถ้าวัดระดับความดันโลหิตได้ค่าตั้งแต่ 130/80 mmhg ถือว่าเป็นภาวะความดันโลหิตสูง |  |  |
| 5 | ถ้าไม่รักษาความดันโลหิตสูง หัวใจจะทำงานหนัก |  |  |
| 6 | เป็นโรคความดันโลหิตสูงนานๆ เส้นโลหิตจะแตกง่าย |  |  |
| 7 | ความรุนแรงของโรคความดันโลหิตสูงทำให้เสียชีวิต |  |  |
| 8 | คนอ้วน หัวใจจะทำงานหนักมากกว่าคนผอม |  |  |
| 9 | ผู้ป่วยความดันโลหิตสูงที่อายุมาก มีโอกาสเกิดอัมพาตได้น้อย |  |  |
| 10 | ในผู้ป่วยความดันโลหิตสูง การดื่มสุราไม่มีผลทำให้เกิดโรคแทรกซ้อน |  |  |
| 11 | โรคความดันโลหิตสูง ไม่ใช่โรคที่อันตราย |  |  |
| 12 | ถ้าปล่อยให้เป็นความดันโลหิตสูงอยู่นานเป็นปี จะทำให้หัวใจโต |  |  |
| 13 | ถ้าความดันโลหิตสูงของท่านลดลงสู่ในระดับปกติ ก็ไม่จำเป็นต้องมาตรวจตามนัด |  |  |
| 14 | ความโกรธ อารมณ์ฉุนเฉียว มีส่วนทำให้ความดันโลหิตสูงขึ้น |  |  |
| 15 | ผู้ป่วยโรคความดันโลหิตสูง ไม่จำเป็นต้องงดสูบบุหรี่ |  |  |
| 16 | ผู้ป่วยความดันโลหิตสูง ควรรับประทานไข่แดงมากๆ |  |  |
| 17 | การลดรับประทานไขมัน ไม่สามารถป้องกันการเกิดเส้นเลือดตีบตัน |  |  |
| 18 | อาหารประเภทตับ เครื่องใน และหนังสัตว์ต่างๆ จัดว่าเป็นอาหารที่มีไขมันมาก |  |  |
| 19 | การรับประทานอาหารพวกแป้ง น้ำตาล น้ำหวาน หรือของหวานมากๆ ไม่ทำให้อ้วน |  |  |
| 20 | รับประทานอาหารที่มีรสเค็ม หมักดอง ไม่ทำให้ความดันโลหิตของท่านสูงขึ้น |  |  |
| 21 | อาหารที่ปรุงจากน้ำมันพืชพวกปาล์มและมะพร้าวทำให้ไขมันในเลือดสูง |  |  |
| 22 | การออกกำลังกายโดยใช้แรงมากๆ เช่น ยกของหนัก แบก หาม จะช่วยลดความดันโลหิตของท่านได้ |  |  |

**ส่วนที่** **3 การรับรู้ความสามารถตนเองต่อการป้องกันโรคความดันโลหิตสูง**

คำชี้แจง โปรดทำเครื่องหมาย / ลงในช่องว่าง ตามความรู้สึก ความคิดเห็น ตามการรับรู้ความสามารถในความเชื่อและผลลัพธ์ที่เกิดขึ้นกับตัวท่านในข้อต่อไปนี้เพียงช่องเดียว คำตอบของท่านไม่มีการตัดสินว่าผิดหรือถูก

| คำจำกัดความ |  |  |  |
| --- | --- | --- | --- |
| (3) | เห็นด้วย | หมายถึง | ท่านเห็นด้วยกับข้อคำถาม |
| (2) | ไม่แน่ใจ | หมายถึง | ท่านไม่แน่ใจกับข้อคำถาม |
| (1) | ไม่เห็นด้วย | หมายถึง | ท่านไม่เห็นด้วยกับข้อคำถาม |

| **ข้อความ** | **ไม่เห็น ด้วย (1)** | **ไม่แน่ใจ2)** | **เห็นด้วย**  **(3)** |
| --- | --- | --- | --- |
| 1. ท่านจะสามารถหลีกเลี่ยงการรับประทานอาหารที่มีส่วนผสมของเกลือ โซเดียม ที่มีการปรุงในอาหาร |  |  |  |
| 2. ท่านจะสามารถหลีกเลี่ยงการกินอาหารที่มีรสชาติเค็ม หมักดอง และรสจัด |  |  |  |
| 3. ท่านจะสามารถหลีกเลี่ยงการรับประทานอาหารที่มีไขมันสูง เครื่องในสัตว์ หรือของทอด |  |  |  |
| 4. ท่านสามารถจะดื่มน้ำอย่างน้อย 7-8 แก้วต่อวัน |  |  |  |
| 5. ท่านจะสามารถรับประทานอาหารที่มีเส้นใยสูง เช่น ผัก ผลไม้ และธัญพืช |  |  |  |
| 6. ท่านจะจัดการผักผ่อนโดยการนอนหลับตอนกลางคืนมากกว่า 7-8 ชั่วโมง |  |  |  |
| 7. ท่านจะมีกิจกรรมผ่อนคลายเครียด เช่น ฟังเพลง ร้องเพลง พบปะเพื่อน หรือ ญาติพี่น้อง |  |  |  |
| 8. ท่านจะมีกิจกรรมทางกายอย่างน้อยวันละ 30 นาที เช่น งานบ้าน ทำไร่ ทำสวน |  |  |  |
| 9. ท่านสามารถจะควบคุมน้ำหนักให้อยู่ในเกณฑ์ปกติ |  |  |  |
| 10. ท่านจะสังเกตอาการผิดปกติ เช่น ปวดศีรษะ คลื่นไส้ อาเจียน |  |  |  |

**ส่วนที่ 4 แบบสัมภาษณ์พฤติกรรมการดูแลสุขภาพของผู้สูงอายุโรคความดันโลหิตสูง (ใช้สัมภาษณ์ทั้งผู้ดูแล และผู้สูงอายุ)**

**คำชี้แจง** แบบสัมภาษณ์ได้ถามถึงพฤติกรรมการดูแลสุขภาพของท่าน หรือการปฏิบัติกิจกรรมใน 4 ด้าน ในเรื่องราวต่าง ๆ ให้กับผู้สูงอายุที่ท่านดูแลหรืออยู่ด้วยในการดำเนินชีวิตประจำวัน ที่ระบุในข้อคำถามแต่ละข้อมากน้อยเพียงใดในช่วง 1 เดือนที่ผ่านมา ให้ผู้สัมภาษณ์อ่านข้อความให้ผู้ถูกสัมภาษณ์ฟังและใส่เครื่องหมาย (/) ในช่องข้อความที่ตรงกับคำตอบของผู้ถูกสัมภาษณ์มาก ที่สุ

| **คำจำกัดความ** |  |  |
| --- | --- | --- |
| (4) | หมายถึง | ปฏิบัติ 5-7 ครั้ง / สัปดาห์ |
| (3) | หมายถึง | ปฏิบัติ 3-4 ครั้ง / สัปดาห์ |
| (2) | หมายถึง | ปฏิบัติ 1-2 ครั้ง / สัปดาห์ |
| (1) | หมายถึง | ไม่เคยปฏิบัติเลย |

| **พฤติกรรม** | **ปฏิบัติ**  **5-7 ครั้ง / สัปดาห์**  **(4)** | **ปฏิบัติ**  **3-4 ครั้ง / สัปดาห์**  **(3)** | **ปฏิบัติ 1-2 ครั้ง / สัปดาห์**  **(2)** | **ไม่เคยปฏิบัติเลย**  **(1)** |
| --- | --- | --- | --- | --- |
| 1. การจัดเมนูการรับประทานอาหารรสเค็ม เช่น ปลาเค็ม ผักกาดดอง บะหมี่กึ่งสำเร็จรูป (มาม่า) |  |  |  |  |
| 2. เติมเครื่องปรุงรส เช่น เกลือป่น น้ำปลา ซีอิ๊วขาว ผงชูรสเพิ่มในอาหาร |  |  |  |  |
| 3. กินอาหารครบ 3 มื้อใน 1 วัน และอาหาร ว่างอย่างน้อย 2 มื้อ (เช้า-บ่าย) |  |  |  |  |
| 4. รับประทานปลาหมึก กุ้ง หอย ปูทะเล เนื้อสัตว์ติดหนังหรือติดมัน เครื่องในสัตว์ อาหารทอด |  |  |  |  |
| 5. รับประทานผลไม้รสหวาน เช่น ทุเรียน มะม่วงสุก ขนุน |  |  |  |  |
| 6. การยืดเหยียดกล้ามเนื้อ เช่น การก้มเอามือแตะพื้น การประสานมือไว้เหนือศีรษะแล้วดันขึ้น การก้มลงเอามือแตะที่พื้น มีประโยชน์ทำให้กล้ามเนื้อและข้อต่อมีความยืดหยุ่น |  |  |  |  |
| 7. การกระตุ้นเคลื่อนไหวในการทำงานบ้าน หรืออาชีพ เช่น ทำความสะอาดบ้าน/ทำสวน/ทำไร่/ทำนา /ขี่จักรยาน หรือเดิน อย่างน้อยวันละ 30 นาที สัปดาห์ละ 5 วัน |  |  |  |  |
| 8. การควบคุมอารมณ์ตนเองได้เมื่อรู้สึกโกรธ เช่น ไม่ขวางปาสิ่งของ ไม่ทำร้ายฝ่ายตรงข้าม ไม่เอะอะโวยวาย |  |  |  |  |
| 9. การควบคุมน้ำหนักให้อยู่เกณฑ์มาตรฐาน |  |  |  |  |
| 10. ดื่มน้ำชา กาแฟ น้ำหวาน น้ำอัดลม |  |  |  |  |
| 11. ดื่มเครื่องดื่มแอลกอฮอล์ในปริมาณน้อย 1-2 แก้วต่อวัน |  |  |  |  |
| 12. ออกกำลังกายอย่างน้อยสัปดาห์ละ 3 วันๆ ละ 30 นาที |  |  |  |  |
| 13. นอนหลับในเวลากลางคืนมากกว่า 7 ชั่วโมง |  |  |  |  |
| 14. มีส่วนร่วมในกิจกรรมของหมู่บ้าน/ชุมชนในการรักษาสิ่งแวดล้อมในชุมชน |  |  |  |  |
| 15. ฝึกสมาธิด้วยการสวดมนต์ |  |  |  |  |
| 16. อ่านฉลากยาก่อนให้ผู้ป่วยรับประทานยาทุกครั้ง |  |  |  |  |
| 17. รับประทานยา หรือสมุนไพรในการลดหรือ  ควบคุมน้ำหนัก |  |  |  |  |
| 18. ให้ผู้ป่วยรับประทานยาลดความดันโลหิตสูงตามจำนวนที่แพทย์สั่ง |  |  |  |  |
| 19. จัดให้ผู้ป่วยจัดการความเครียด อารมณ์ เช่น ทำบุญ/พักผ่อน หรือทำกิจกรรมร่วมกันกับสมาชิกในครอบครัว เช่น กินอาหาร /ดูทีวี/ ฯลฯ |  |  |  |  |
| 20. กระตุ้นให้ผู้ป่วยทำกิจวัตรประจำวันด้วยตนเอง เช่น แปรงฟัน อาบน้ำ ล้างมือ รับประทานอาหาร |  |  |  |  |

**………………………ขอบคุณทุกท่านที่ให้ความร่วมมือในการตอบคำถาม.............................................**
