# Supplementary material for: Effects of a social support family caregiver training program on changing blood pressure and lipid levels among elderly at risk of hypertension in a northern Thai community
Source: PLoS One. 2021 Nov 30;16(11):e0259697. doi: 10.1371/journal.pone.0259697 (PMC8631627; doi:10.1371/journal.pone.0259697)
Supplement: S2 File — (DOCX) [file pone.0259697.s002.docx]

Questionnaires

Part 1: (1.1) General characteristics of Elderly

Note: please mark √ for your answers

| **Variables** |  | | |
| --- | --- | --- | --- |
| 1. age | ……………………………………….years | | |
| 2. Gender | ( ) 1. male | ( ) 2. Female | |
| 3. Education level | ( ) 1. No education | ( ) 2. Primary school | |
|  | ( ) 3. Secondary school | ( ) 4. Bachelor or above | |
| 4. Marital status | ( ) 1. Single/Never Married | ( ) 2. Married | |
|  | ( ) 3. Divorced | ( ) 4. Separated | |
|  | ( ) 5. Widowed | ( ) 6. Others…………….. | |
| 5. Working status | ( ) 1. Not working | ( ) 2. Currently working | |
|  |  |  | ( ) 2.1 General employment |
|  |  |  | ( ) 2.2 Agriculture |
|  |  |  | ( ) 2.3 Trade |
|  |  |  | ( ) 2.4 Others……… |
| 6. Income | ( ) 1. Insufficient | ( ) 2. Sufficient | |
| 7. Dietary habit/tasty food | ( ) 1. No( ) 3. Sugar( ) 5. Others……… | ( ) 2. Salt( ) 4. Fat | |
| 8. Alcohol drinking | ( ) 1. No | ( ) 2. Yes | |
| 9. Smoking | ( ) 1. No | ( ) 2. Yes | |
| 10. Body mass index (BMI) | ………………………………………. kg/m2 | | |
| 11. Receiving information about HT | ( ) 1. No | ( ) 2. Yes ( ) 2.1 By public health officer  ( ) 2.2 By health volunteer  ( ) 2.3 By online media ( ) 2.4 Others……… | |
| 12. Total cholesterol | .................................. mg/dl | | |
| 13. Blood pressure | ............................. mmhg | | |

(1.2) General characteristics of caregiver

Note: please mark √ for your answers

| **Variables** |  | | |
| --- | --- | --- | --- |
| 1. age | ……………………………………….years | | |
| 2. Gender | ( ) 1. male | ( ) 2. Female | |
| 3. Education level | ( ) 1. No education | ( ) 2. Primary school | |
|  | ( ) 3. Secondary school | ( ) 4. Bachelor or above | |
| 4. Marital status | ( ) 1. Single/Never Married | ( ) 2. Married | |
|  | ( ) 3. Divorced | ( ) 4. Separated | |
|  | ( ) 5. Widowed | ( ) 6. Others…………….. | |
| 5. Working status | ( ) 1. Not working | ( ) 2. Currently working | |
|  |  |  | ( ) 2.1 General employment |
|  |  |  | ( ) 2.2 Agriculture |
|  |  |  | ( ) 2.3 Trade |
|  |  |  | ( ) 2.4 Others……… |
| 6. Income | ( ) 1. Insufficient | ( ) 2. Sufficient | |
| 7. Relationship to patient with HT | ( ) 1. Spouse( ) 3. Grandchild | ( ) 2. Child ( ) 4. Others………………… | |
| 8. Alcohol drinking | ( ) 1. No | ( ) 2. Yes | |
| 9. Smoking | ( ) 1. No | ( ) 2. Yes | |
| 10. Receiving information | ( ) 1. No | ( ) 2. Yes ( ) 2.1 By public health officer  ( ) 2.2 By health volunteer  ( ) 2.3 By online media  ( ) 2.4 Others……… | |

Part 2: Knowledge toward health care for elderly patients with hypertension

Note: please mark √ for your answers

| **Items** | **Contents** | **Yes** | **No** |
| --- | --- | --- | --- |
| 1. | High blood pressure disease, resulting in abnormal aorta. |  |  |
| 2. | High blood pressure may have headaches, shortness of breath or nosebleeds. |  |  |
| 3. | High blood pressure cannot be treating; it can lead to vision impairment. |  |  |
| 4. | Blood pressure level from 130/80 mmhg; It is high blood pressure that affects the illness. |  |  |
| 5. | Long-term high blood pressure disease will result in blood clots easily. |  |  |
| 6. | High blood pressure has reached a severe or life-threatening stage. |  |  |
| 7. | High blood pressure can cause hardening and thickening of the arteries (atherosclerosis), which can lead to a heart attack, and stroke. |  |  |
| 8. | High blood pressure can cause weakened and narrowed blood vessels in your kidneys. |  |  |
| 9. | People who ate a lot of salt were more likely to develop high blood pressure than those who ate a lot of salt. |  |  |
| 10. | Females develop high blood pressure more frequently than males. |  |  |
| 11. | Having overweight or obesity increases your risk for high blood pressure. |  |  |
| 12. | The risk of high blood pressure increases as you age. Until about age 64. |  |  |
| 13. | In hypertensive patients, drinking alcohol does not cause complications. |  |  |
| 14. | Patients with high blood pressure do not need to quit smoking. |  |  |
| 15. | High levels of stress can lead to a temporary increase in blood pressure. |  |  |
| 16. | Patients with high blood pressure should choose to eat foods that are high in protein. |  |  |
| 17. | Patients with high blood pressure should not eat fruits and vegetables to increase blood sugar. |  |  |
| 18. | Hypertensive patients eating salty food will not raise your blood pressure. |  |  |
| 19. | Physical activity can help keep you at a healthy weight and lower your blood pressure. |  |  |
| 20. | Patients with high blood pressure should exercise at least 2 hours a day such as brisk walking, doing housework, or bicycling. |  |  |
| 21. | Getting enough sleep is part of keeping your heart and blood vessels healthy. |  |  |
| 22. | Patients with high blood pressure when suffering from illness such as nausea, vomiting should seek medical attention. |  |  |

Part 3: Self-efficacy toward the prevention of hypertension

Note: please mark √ for your answers.

| **Note;** |  |  |
| --- | --- | --- |
| (3) | = | Agree |
| (2) | = | Not sure |
| (1) | = | Disagree |

| **Content** | **Disagree** | **Not sure** | **Agree** |
| --- | --- | --- | --- |
| 1. You will be able to avoid eating foods that contain sodium salt that is cooked in food. |  |  |  |
| 2. You will be able to avoid eating salty, pickled, and spicy foods. |  |  |  |
| 3. You will be able to avoid eating foods that are high in fat, offal, or fried. |  |  |  |
| 4. You will be able to drink water at least 7-8 per day. |  |  |  |
| 5. You will be able to eat foods that are high in fiber such as vegetables, fruits, and whole grains. |  |  |  |
| 6. You will manage your rest by sleeping more than 7-8 hours at night. |  |  |  |
| 7. You will have stress-relieving activities such as listening to music, singing, meeting friends or relatives. |  |  |  |
| 8. You will have at least 30 minutes of physical activity each day, such as housework, farming, gardening. |  |  |  |
| 9. You will be able to control your weight to be within the normal range. |  |  |  |
| 10. You will notice unusual symptoms such as headache, nausea, and vomiting. |  |  |  |

Part 4: Behaviors toward health care for elderly patients with hypertension

Note: please mark √ for your answers. The interview questionnaire asked about your health care behavior or the activities performed by the elderly you care for or live with in your daily life. It asked the elderly care behavior of caregiver in the past 1 month.

| **Note;** |  |  |
| --- | --- | --- |
| (4) | = | Regularly (5-7 times / weeks) |
| (3) | = | Sometimes (3-4 times / weeks) |
| (2) | = | Rarely (1-2 times / weeks) |
| (1) | = | Never |

| Behaviors | | Regularly  (4) | Sometimes  (3) | Rarely  (2) | Never  (1) |
| --- | --- | --- | --- | --- | --- |
| 1. | As you eat, you will add fish sauce, salt, or seasonings to your food. |  |  |  |  |
| 2. | Eat sweet fruits such as durian, ripe mango and jackfruit. |  |  |  |  |
| 3. | Eat seafood such as squid, shrimp, clams, and crabs. |  |  |  |  |
| 4. | Eat fatty foods, offal, and fried foods. |  |  |  |  |
| 5. | Eat 3 full meals a day and eat at least 2 snacks (morning-afternoon). |  |  |  |  |
| 6. | Drink at least 7-8 glasses of water a day. |  |  |  |  |
| 7. | Stress management such as meditation, going to temples, making merit. |  |  |  |  |
| 8. | Exercise or physical activity |  |  |  |  |
| 9. | Drink small amounts of 1-2 alcoholic beverages per day. |  |  |  |  |
| 10. | Keeping your weight up to the standard |  |  |  |  |
| 11. | Exercise (walking or biking 30 min per day) |  |  |  |  |
| 12. | Physical activity activities (household work, farming, and gardening); |  |  |  |  |
| 13. | Sleeps more than 7 hours at night |  |  |  |  |
| 14. | Drink tea, coffee, syrup, soft drinks. |  |  |  |  |
| 15. | Practice meditation by paying respect to monks and chanting |  |  |  |  |
| 15. | Read drug labels before giving patients a prescription. |  |  |  |  |
| 17. | Taking medication, herbal medicine to reduce, or control weight. |  |  |  |  |
| 18. | You care for them about taking high blood pressure medication according to your doctor's treatment. |  |  |  |  |
| 19. | You encourage them to do daily activities by themselves, such as brushing their teeth, showering, washing their hands, eating. |  |  |  |  |
| 20. | When they have abnormal symptoms, you will be taken to the hospital to see your doctor. |  |  |  |  |
