# Supplementary material for: Effects of a social support family caregiver training program on changing blood pressure and lipid levels among elderly at risk of hypertension in a northern Thai community
Source: PLoS One. 2021 Nov 30;16(11):e0259697. doi: 10.1371/journal.pone.0259697 (PMC8631627; doi:10.1371/journal.pone.0259697)
Supplement: Attachment — Submitted filename: Response to Reviewer-KS-26092021.docx [file pone.0259697.s004.docx]

School of Public Health, University of Phayao

19/2 Mae-Ka, Meung district, Phayao Province 56000, Thailand

Tel.(66 54 46 6666

September 25, 2021

Dear Sir/Madam,

According to submit the manuscript title ““Effect of a Social Support Family Caregiver Training Program on Changing Blood Pressure and Lipid Levels Among Elderly with Hypertension Risk in Northern Thai Community” PONE-D-21-22371 - [EMID:66137e20164778db].”. We’ve improved according to the reviewer's recommendations below are the following;

1. **Journal Requirements:**

| **Journal Requirements** | **Revised** |
| --- | --- |
| 1. Please ensure that your manuscript meets PLOS ONE's style requirements, including those for file naming. The PLOS ONE style templates can be found at  -https://journals.plos.org/plosone/s/file?id=wjVg/PLOSOne  _formatting_sample_main_body.pdf and  -https://journals.plos.org/plosone/s/file?id=ba62/  PLOSOne_formatting_sample_title_authors_affiliations.pdf | We have revised and improved accordingly to those forms. |
| 2. Thank you for submitting your clinical trial to PLOS ONE and for providing the name of the registry and the registration number. The information in the registry entry suggests that your trial was registered after patient recruitment began. PLOS ONE strongly encourages authors to register all trials before recruiting the first participant in a study.  As per the journal’s editorial policy, please include in the Methods section of your paper:  1) your reasons for your delay in registering this study (after enrolment of participants started);  2) confirmation that all related trials are registered by stating: “The authors confirm that all ongoing and related trials for this drug/intervention are registered”. | Thank you very much for your kind review.  1. The reason for the delay in the registration of the participants is because the TCTR registration system has undergone major renovations to upgrade the website during November 2020 to January 2021.  (https://www.thaiclinicaltrials.org/public/download/  announce/announcement.pdf)  In which our previous research process has passed the University of Phayao Human Ethics Committee Certification ID:2/089/61. The process of the accreditation of research projects in accordance with international standards of human research ethical guidelines include: Declaration of Helsinki, The Belmont Report, CIOMS Guideline, and International Conference on Harmonization in Good Clinical Practice (ICH-GCP).  Therefore, in this study “The authors confirm that all ongoing and related trials for this drug/intervention are registered”. |
| 3. Please include additional information regarding the survey or questionnaire used in the study and ensure that you have provided sufficient details that others could replicate the analyses. For instance, if you developed a questionnaire as part of this study and it is not under a copyright more restrictive than CC-BY, please include a copy, in both the original language and English, as Supporting Information. | Thank you very much for your kind review.  We have put a copy of both original Thai and English versions in Supporting information as showed in line 182-183;  -S1 Questionnaire. Questionnaire in the Thai language.  -S2 Questionnaire. Questionnaire in the English language. |
| 4. Thank you for stating the following financial disclosure:  “The authors are grateful to the research project was supported by the Thailand science research and innovation fund and the University of Phayao the Unit of Excellence named “Health Promotion and Quality of Life” grant number FF64-UoE009.”  - Please state what role the funders took in the study. If the funders had no role, please state: "The funders had no role in study design, data collection and analysis, decision to publish, or preparation of the manuscript."  If this statement is not correct you must amend it as needed.  Please include this amended Role of Funder statement in your cover letter; we will change the online submission form on your behalf. | Thank you very much for your kind review.  In this study, the funders had no role in process: "The funders had no role in study design, data collection and analysis, decision to publish, or preparation of the manuscript." |
| 5. Thank you for stating the following in the Acknowledgments Section of your manuscript:  “The authors are grateful to the research project was supported by the Thailand science research and innovation fund and the University of Phayao the Unit of Excellence named “Health Promotion and Quality of Life” grant number FF64-UoE009.”  We note that you have provided funding information within the Acknowledgements Section. Please note that funding information should not appear in the Acknowledgments section or other areas of your manuscript. We will only publish funding information present in the Funding Statement section of the online submission form.  Please remove any funding-related text from the manuscript and let us know how you would like to update your Funding Statement. Currently, your Funding Statement reads as follows:  “The authors are grateful to the research project was supported by the Thailand science research and innovation fund and the University of Phayao the Unit of Excellence named “Health Promotion and Quality of Life” grant number FF64-UoE009.”  Please include your amended statements within your cover letter; we will change the online submission form on your behalf. | Thank you very much for your kind review.  We have removed the funding information from the Acknowledgements Section at your suggestion.  We wish it to be written as follow;  “The authors are grateful to the research project was supported by the Thailand science research and innovation fund and the University of Phayao the Unit of Excellence named “Health Promotion and Quality of Life” grant number FF64-UoE009.” |
| 6. We note that you have indicated that data from this study are available upon request. PLOS only allows data to be available upon request if there are legal or ethical restrictions on sharing data publicly. For information on unacceptable data access restrictions, please see http://journals.plos.org/plosone/s/data-availability#loc-unacceptable-data-access-restrictions.  In your revised cover letter, please address the following prompts:  a) If there are ethical or legal restrictions on sharing a de-identified data set, please explain them in detail (e.g., data contain potentially identifying or sensitive patient information) and who has imposed them (e.g., an ethics committee). Please also provide contact information for a data access committee, ethics committee, or other institutional body to which data requests may be sent.  b) If there are no restrictions, please upload the minimal anonymized data set necessary to replicate your study findings as either Supporting Information files or to a stable, public repository and provide us with the relevant URLs, DOIs, or accession numbers. Please see http://www.bmj.com/content/340/bmj.c181.long for guidelines on how to de-identify and prepare clinical data for publication. For a list of acceptable repositories, please see http://journals.plos.org/plosone/s/data-availability#loc-recommended-repositories.  We will update your Data Availability statement on your behalf to reflect the information you provide. | Thank you very much for your kind review.  We are happy to share information publicly.  -S3 Dataset. In line 300-301. |

2. **Reviewers' Comments to Author**

| **Reviewers' comments and suggestions** | **Revised** |
| --- | --- |
| **Reviewer 1** |  |
| 1. This randomized interventional study evaluates the effects of a Social Support Family Caregiver Training Program (SSFCTP) on changing blood pressure and lipid levels among elderly at risk of hypertension. This is an interesting study. | Thank you very much for your kind review. |
| 2. My major concern is that in intervention arm, the authors conducted not only SSFCTP but also weight management, alcohol consumption, smoking, physical activity, taking medication, BP and cholesterol control, and self-regulation. How the authors purify the SSECTP effect in this setting? | In this study, we use the basis of the self-regulation concept of activities where they are given a diary to record their behavior with caregivers and elders together.  In addition, in the course of our activities we will keep track of and check the note taking to avoid forgetting as showed in line 546-551. |
| **Reviewer 2** |  |
| 1. This paper was a clinical trial study in a northern Thai community. This study was showed that elderly people at risk of hypertension increased knowledge about hypertension using SSFCTP and importance of caregivers.  2. Although this manuscript is interesting and meaningful, there are some concerns in it. | Thank you very much for your kind review. |
| Major comments  1) The most serious concern is a short-term study in 12 weeks.  We would like to know odds ratio or hazard ratio for morbidity of hypertension. Increasing knowledge about hypertension keeps blood pressure good, and prevent of hypertension will be make it decreased. This paper should focus on blood pressure. | We have revised according to your suggestion. We used crosstabs to calculate the risk ratio (RR) for hypertension at each time point that may be appropriate for prospective data collection. The risk of hypertension in the intervention group decreased significantly despite the 12-week study in page 12-13 (line 298-300) and page 19 (line 444-446). |
| 2) Are there any clinical implications in your study? I think SSFCTP is a meaningful program. But SSFCTP has several contents such as knowledge, efficacy, and behavior. Which content was a most important for health and blood pressure? | In this study, The SSFCTP program were effective for behavior, blood pressure and lipid levels change in elderly.  In the process of conducting research activities, we have used many content to provide activities to enhance skills of the elderly.  Specifically, self-care behaviors concerning dietary behaviors they must be monitored by behavioral. In addition, the use of a caregiver model to support, encourage self-care among older adults will result in them participating in regulating blood pressure and lipid levels. We have presented the discussion in line 551-556. |
| 3) Table4 and Table5 were so complicated. These tables should be represented in figures as you emphasized variables. | Table 5 have been removed to simplify the presentation of the results. However, Table 4 is kept because the values of each variable in the intervention and control groups should be clearly visible. |
| Minor comments  In line at 477, the paper was written TSSFCP. Does it mean SSFCTP? | We have edited according to your recommendation in line 467. |
| **Reviewer 3** |  |
| Present study corresponds to two arm parallel group randomized controlled trial. I have several reservations about the study which needs further explanations. | Thank you very much for your kind review. |
| 1. It looks like two groups (treatment and control) are collected from two entirely different village/region.  - This mean the effect due to particular location cannot be eliminated. This is true randomization authors must explain why this is done and the possible weakness of the findings. | Because intervention and control groups are collected from two entirely different village to avoid any potential contamination, we called the quasi-experimental study. However, due to their proximity and random method used to select participants in each group, both groups shared similar characteristics. It's plausible that this might have no or only a minor effect on our findings in line 112-116. |
| 2. Elderly and caregiver are they belong to the same family, or they are chosen at random is not clear. If they are form same family they from a dyad which must be accounted for during statistical modelling. | Elderly and caregiver are members of the same family. However, there was no statistically significant difference in the factor "relationship to the patient" between the intervention and control groups. So, we did not account for this variable. |
| 3. The most serious concern is the power analysis presented in the paper (line 134-140). It states a two-sample t-test is used to power the study. Yet the main analysis proposed is RMANOVA.  Repeated measure ANOVA is the correct model for longitudinal design, however, same model must be used for power analysis, which is not done.  The paper also report pre-post type design for which t-test can be used for power analysis, but then RMANOVA should not be used for data analysis.  More importantly region effect and dyad effect (if true) should be part of the model building. From the table 3 reporting it is clear RMANOVA is the right model but the study is not powered properly. | Thank you very much for your concern. To assess the program's effectiveness, we used both repeated measure ANOVA and the t-test. Although the t-test was used to calculate sample size, it was found that this number provided sufficient power for the study (>80%) after computing to achieve power from RMANOVA based on blood pressure as a primary outcome of this study. After that, we would like to show the RMANOVA results. We did not consider the region effect because each participant group was only conducted for one village, and it was found that baseline characteristics between two groups were not different. |
| 4. What about missing data and multiple testing issues, which is not addressed sufficiently. Please clearly stated which one is/are primary outcome/s. In table 3 multiple interaction term is tested, which means multiple testing correction must be used as otherwise type-1 error rate will be inflated. This may indicate the study is under-powered. | The missing data was not found in this study. Blood pressures are a primary outcome of this study. Also, we calculated to achieve power from RMANOVA for within-between interaction and found that our sample size provided sufficient power (>80%) in line 298-300. |

We have included acknowledgments, conflicts of interest, and funding source after discussion.

If you need further information, please do not hesitate to contact me.

Thank you for kindness.

Sincerely,

Corresponding author

(Katekaew Seangpraw)

Email: eungkaew@gmail.com

Mobile Phone: (66) 614428988

School of Public Health, University of Phayao, Phayao, Thailand
